# National Chronic Obstructive Pulmonary Disease (COPD) Awareness Month — November 2014

**Published:** 2014-11-21

Chronic obstructive pulmonary disease (COPD) is a respiratory condition that makes it hard to breathe by limiting airflow in and out of the lungs. COPD includes emphysema and chronic bronchitis. Each year, more persons in the United States die from COPD than from stroke, injuries, or diabetes (1). The symptoms of COPD include frequent coughing (sometimes called “smoker’s cough” if the patient is a current or former smoker), excess phlegm or sputum production, shortness of breath while doing activities the patient used to be able to do, wheezing, and not being able to take a deep breath. The primary cause of COPD in the United States is smoking, but one fourth of patients with COPD have never smoked (2). The risk for COPD increases with age and is higher among women than men and among American Indians/Alaska Natives than other ethnic groups (3).

November is National COPD Awareness Month. The observance is supported by the National Heart, Lung, and Blood Institute’s COPD: Learn More, Breathe Better campaign. This year, the campaign encourages persons who are experiencing COPD symptoms to “Take the First Step” and discuss their symptoms with their physician. Lung function can be evaluated through a simple breathing test called spirometry. Although COPD currently has no cure, it can be treated, making it possible for patients to improve their quality of life.

More information about COPD is available from CDC at http://www.cdc.gov/copd and from the National Heart, Lung, and Blood Institute at http://www.nhlbi.nih.gov/health/educational/copd.
